# *Chlamydia trachomatis* screening in urine among asymptomatic men attending an STI clinic in Paris: a cross-sectional study

**DOI:** 10.1186/s12879-018-3595-6

**Published:** 2019-01-08

**Authors:** Paul Rondeau, Nadia Valin, Dominique Decré, Pierre-Marie Girard, Karine Lacombe, Laure Surgers

**Affiliations:** 1 Department of Infectious Diseases, University Hospital Saint-Antoine, APHP, 75012 Paris, France; 2 Department of Bacteriology, University Hospital Saint-Antoine, APHP, 75012 Paris, France; 3 Sorbonne University, UPMC Univ Paris 06 CR7, Paris, France; INSERM U1135, CIMI, Team E13, 75012 Paris, France; 4 Sorbonne University, UPMC Univ Paris 06, UMR_S 1136, Institut Pierre Louis d’Epidémiologie et de Santé Publique, F-75013 Paris, France

**Keywords:** *Chlamydia trachomatis*, Screening men urine

## Abstract

**Background:**

The incidence of *Chlamydia trachomatis* (Ct) urethritis has been increasing for the past 10 years. There is little data regarding the screening of Ct infection in asymptomatic men in France, despite the national recommendation to screen at-risk asymptomatic men under 30 attending Sexually Transmitted Infections (STI) clinics. Recent data from the French surveillance network Rénachla show indeed that systematic screening is still focused on women. The objective of our study was to determine the prevalence and risk factors for Ct infection in asymptomatic men under 30 attending an STI clinic located in Paris, France.

**Methods:**

We performed a cross-sectional study between April 4, and December 31, 2016 in the database of the software DIAMM Client V8 used in our STI clinic. We extracted the demographic characteristics, sexual behavior and result of STI screening of all asymptomatic men who had consulted and given their consent for the use of their personal data. Those data were collected in usual care through a standardized questionnaire filled in during an appointment with a trained physician. STI screening was performed using PCR kit CT/NG Abbott Realtime® on first void urines. For MSM, a rectal swab was also collected. Risk factors for Ct infection were analyzed by univariate and multivariate modeling using STATA software 8.2.

**Results:**

Among 872 men who had attended the clinic, 647 were included and 37 (5.7, 95% CI 4.2 to 7.8) were positive for Ct in urine. In univariate analysis, men who had unprotected sex in the last 6 weeks (OR 2.40 (95%CI 1.16 to 4.94), *p* = 0.02), and those who had an infected partner (OR 7.6 (95%CI 3.03 to 20), *p* = 0.0001) were more likely to be infected. In the multivariate analysis having an infected partner was the only risk factor (OR 11.1(95% CI 3.7 to 33.3), p = 0.0001) that remained significant.

**Conclusion:**

Prevalence of Ct infection is high among asymptomatic men of 30 years or less attending our urban STI clinic especially among those with an infected partner. The Ct screening among this population associated with partner notification, as recommended by the French national guidelines, should be more widely implemented.

## Background

*Chlamydia trachomatis* (Ct) is an obligate intracellular bacterium responsible for the most commonly reported sexually transmitted infection (STI) in Europe where its incidence has increased by 68% between 2004 and 2013 [1, 2]. In France, its prevalence has been estimated to be 3.2% among women in 2006 and 2.5% among sexually active men aged 18 to 29 years [3]. In 2012, the incidence was estimated to be 257 per 100,000 persons aged 15 to 49 years [4]. Between 2013 and 2015, the number of Ct infections registered by the French surveillance network Rénachla (Réseau National Chlamydia) increased by 19% in men and 8% in women [5]. The positivity rate of tests in this network was 6.5% in men and women in 2015.

Most European countries have implemented an opportunistic screening of young women but screening also high-risk men could be cost-saving [2, 6]. In France there is an opportunistic systematic screening of at-risk population attending STI clinics, abortion and family planning centers. In 2003, the Agence Nationale d’Accréditation et d’Evaluation en Santé (ANAES), suggested to screen men under 30 in addition to women under 25 years old in these facilities and called for epidemiological studies before extending the screening to the general population [7]. However, few studies have been published since then. Recent data from Rénachla also suggest that screening in France is still focused on women (64% of reported cases) and systematic screening is decreasing [5]. In order to provide current data to inform about the need for systematic screening of men, an epidemiological study was conducted on medical records of the STI clinic of Saint-Antoine University teaching hospital, Paris. Our objective was to determine the prevalence and risk factors of Ct urogenital infection in asymptomatic men under 30 attending the STI clinic.

## Methods

### Setting, patients and data collection

We conducted a cross-sectional study among all asymptomatic men under 30 who had attended the STI clinic from April 4 to December 31, 2016, using the database of the software DIAMM Client V8. Those data were collected through a standardized questionnaire filled with the help of a trained physician during a face-to-face interview. The questionnaire was elaborated based on evidence from the literature and enquired about demographic data (age, origin, level of education, health coverage) and characteristics of sexual behavior (sexual orientation, number of partners in the last 12 months, date of last unprotected intercourse, sexual intercourse with regular or occasional partners, history of STI) [1, 3, 8, 9].

### Screening for Ct

Screening was systematically performed by PCR kit CT/NG Abbott Realtime® on a first-void urine sample collected with consent after the consult of every asymptomatic male under 30 attending the STI clinic. For MSM, we also collected a rectal swab. Screening for other STI was performed according to the usual practice. Results were communicated to the participants 7 days later during a clinical appointment. Patients with positive Ct PCR were treated by doxycycline (100 mg × 2 for 7 days). Treatment for other STI were given when necessary. Partner notification was encouraged.

### Ethics, consent and permissions

This cross-sectional study was conducted upon a database of patients treated according to standard care under the MR04 methodology (registration number 1809221118); therefore no ethics approval was necessary according to French law (http://www.legifrance.gouv.fr/eli/decret/2017/5/9/AFSP1706303D/jo/texte). However, informed consent for the collection, storage and use of personal data for scientific studies was sought from each patient. The database was declared to the Comission Nationale de l’Informatique et des Libertés (CNIL).

### Statistical analysis

The population was described using medians and interquartiles (IQ25–75) for quantitative variables. The prevalence of Ct infection was estimated by the number of positive results in the total number of screened individuals, and expressed with its confidence interval (95% CI) calculated using the exact binomial method. Groups were compared by the chi-square or Fisher’s exact test, as appropriate. A logistic regression was used to determine the risk factors for Ct infection among the variables with a *p* < 0.20 in the univariate analysis. *P* values below 0.05 were considered significant. Univariate and multivariate analysis were performed using STATA 8.2 software.

## Results

During the study period, 872 men under 30 years old attended the center and 225 (25.8%) could not be included for the following reasons: 189 (21.7%) did not sign the consent form, 36 (4.1%) were symptomatic. Therefore, 647 (74.2%) individuals were analyzed. Their characteristics are described in Table 1.

**Table 1:**
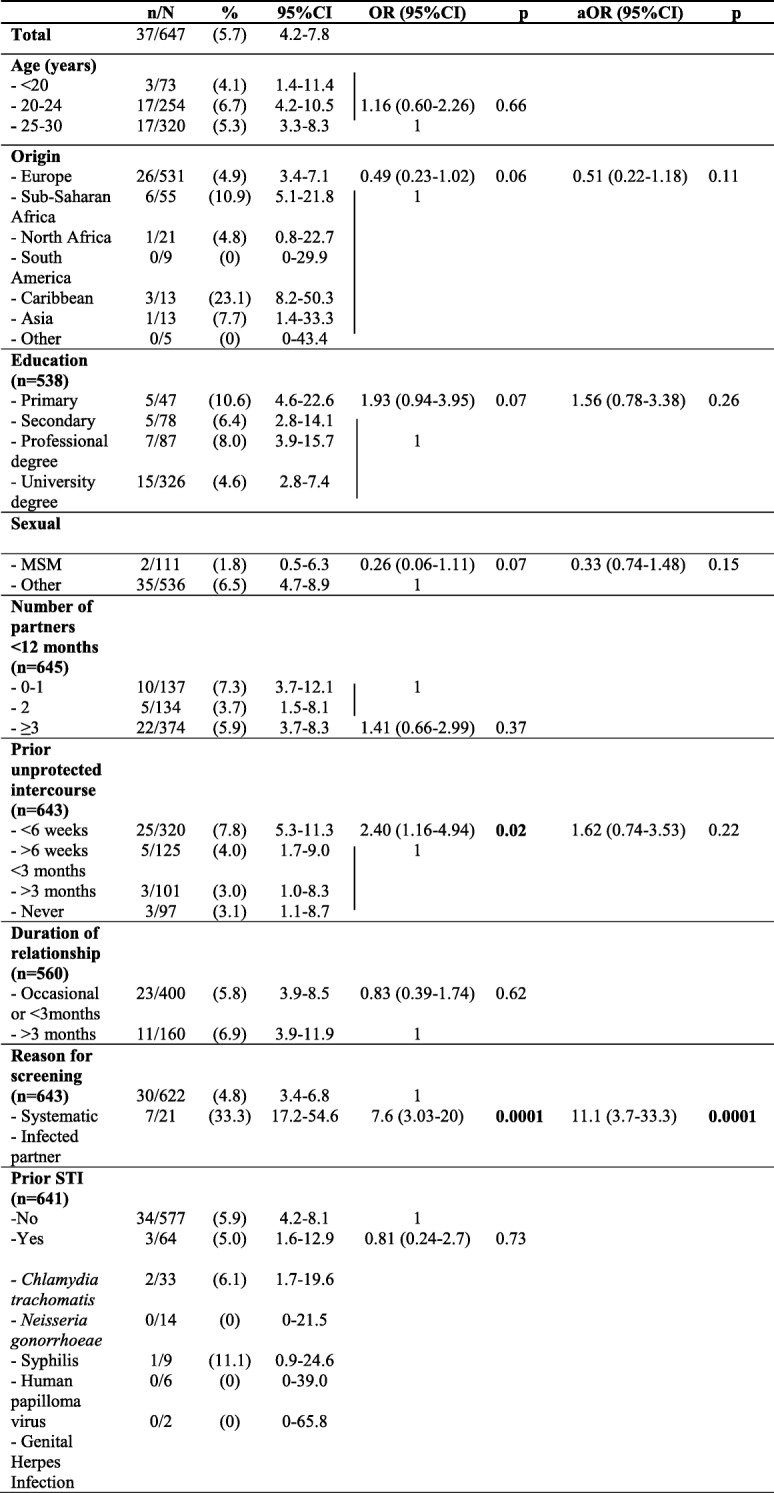
Risk factors for *Chlamydia trachomatis* infection

Bold fonts indicate a statistically significant p value

N is the total number of participants tested in each subgroup

n is the number of participants with a positive PCR in each subgroup

MSM Men who have Sex with Men

OR Odds Ratio

aOR adjusted Odds Ratio

The population included in the study had a median age of 24 (22–26) years. They were mostly Europeans (*n* = 537/647, 82.1%), highly educated (*n* = 326/538, 60.6%), self-declared heterosexuals (*n* = 538/647, 82.8%), with a median of 3 (2–5) partners in the last 12 months.

Prevalence of Ct in urine samples was 5.7% (*n* = 37/647, 95% CI 4.2 to 7.8). It reached a peak in the reference group of men between 20 to 24 years old (*n* = 17/254, 6.7, 95% CI 4.2 to 10.5) and remained high in men aged 25 to 30 years old (n = 17/320, 5.3, 95% CI 3.3 to 8.3). Men from the positive group had a median age (IQR) of 24 (22–27) years and a median of 3 (1–6) partners in the last 12 months. Their median age of first intercourse was 18 (17–21) years old. Twenty-two declared being vaccinated against hepatitis B (*n* = 22/37, 59.5%).

In the univariate analysis, men who tested positive had had significantly more unprotected sex in the last 6 weeks (OR 2.40 (95%CI 1.16 to 4.94), *p* = 0.02), and had more often an infected partner (OR 7.6 (95%CI 3.03 to 20), *p* = 0.0001). Infected men tended to be non-European and not MSM with a lower educational status, but the difference was not statistically significant. Having an infected partner was the only risk factor that remained significant in the multivariate analysis (OR 11.1(95% CI 3.7 to 33.3), p = 0.0001).

There was a positivity rate of (13/111, 17.1%) among the rectal swabs we collected in MSM. Three men were coinfected with Ct and *Neisseria gonorrhoeae* (Ng) (*n* = 3/647, 0.5%). Five active cases of hepatitis B infections (*n* = 5/647, 0.8%), two cases of HIV infections (*n* = 2/647, 0.5%), two cases of syphilis (n = 2/647, 0.5%) and one case of chronic hepatitis C (*n* = 1/647, 0.2%) were also diagnosed. Among the 647 included individuals, 517(79.9, 95% CI 76.6 to 82.9) came back for their follow-up appointment and were treated. Two patients with positive results did not come back to get their results.

## Discussion

A high prevalence (5.7, 95% CI 4.2 to 7.8) of Ct infection was found in asymptomatic men under 30 attending the STI clinic of our university teaching hospital located in Paris, France. The main risk factor for Ct infection was having an infected partner.

The population of the study was similar to the one attending the center the previous year in terms of origin (82.1 vs. 80% Europeans) and sexual orientation (17.2 vs. 18.1% MSM).

This prevalence observed in a high risk population attending an STI clinic is unsurprisingly higher than the 2.5% prevalence reported in 2006 in the French national study Natchla among 18–29 years old sexually experienced men from the general population [3]. In the national study, men had a lower median number of partners in the last 12 months than in our study (1 vs. 3). The prevalence found in our study is also higher than that of 4.4% reported in the Chlamyweb study among sexually active French men between 18 to 24 years old in 2012 contacted through the internet for a home-based self-sampling screening [8]. In this study men were younger and had fewer partners in median. These two studies (Natchla and Chlamyweb) were conducted nationwide whereas ours took place in the Ile-de-France region which is known for its high incidence of Ct [3, 4]. Our prevalence was closer though slightly inferior to the one reported by Clarivet et al who found a prevalence of Ct of 7.1% in asymptomatic men attending an STI clinic in Montpellier in 2009 [10]. The prevalence in our study was highest in men between 20 to 24 years old and remained high between 25 to 30 years old. Similar age distribution has been described before and was also found in the French national study Natchla, and in the surveillance network Rénachla [3, 5]. These data support the need of a better implementation of the systematic screening of asymptomatic men under 30 attending STI clinics in France.

The main risk factor identified here was having an infected partner, which was expected. Unprotected sex in the last 6 weeks also seemed to be associated with Ct infection in the univariate analysis which is concordant with previous data from the literature, however it did not remain significant in the multivariate analysis [9]. Men having sex with men (MSM) tended to be less represented in the positive group in the univariate analysis. This could be because they have access to a regular screening and treatment by their personal physician or in community settings. On the contrary, the number of partners or the status of “occasional relationship” of the last partner did not appear to be associated to a positive Ct result unlike in other studies [3, 8]. This may be because all patients in our center are already at high risk of STI and therefore identifying these risk factors would have required more statistical power. These data underline if needed be, that having an infected partner is a major risk factor. When expanding the systematic screening of men particular attention should be paid to partner notification.

Our study, conducted with a cross-sectional design on a relatively large number of patients, is one of the few to provide a current description of the population of men eligible for Ct screening in France. It does nevertheless present some limitations. Given the high-risk profile for STI of the study population, the results could be extended to the population attending other STI clinics, but not to the general population. There are around 15% missing data for two variables. It is partly due to language barrier despite the possibility to call a professional translator when necessary. The questionnaires were filled in during a face-to-face interview, which might have led to incomplete disclosure of sexual behavior due to social desirability bias. These limitations may have contributed to a lack of statistical power to better discriminate risk factors for Ct infection.

## Conclusion

This cross-sectional study reports a high current prevalence of Ct infection in asymptomatic men attending an STI clinic for voluntary screening and partner infection is the main risk factor. The systematic screening of at-risk men under 30 attending STI clinics with partner notification as recommended by national guidelines should be more widely implemented.

## Abbreviations

Ct: *Chlamydia trachomatis*
STI: Sexually Transmitted Infection
